# The Role of Mobility in HIV Intervention Engagement and Community Mobilization among Female Sex Workers Living with HIV in the Dominican Republic

**DOI:** 10.1007/s10461-026-05053-1

**Published:** 2026-02-17

**Authors:** Maria De Jesus, Eric R. Schuler, Julia Rivara, Noya Galai, Clare Barrington, Yeycy Donastorg, Martha Perez, Hoisex Gomez, Deanna Kerrigan

**Affiliations:** 1 Department of Environment, Development, and Health, School of International Service, American University, Washington, DC, USA; 2 Center on Health, Risk, and Society, American University, Washington, DC, USA; 3 Center for Teaching, Research & Learning, American University, 20016 Washington, DC, USA; 4 Department of Development Management, School of International Service, American University, Washington, DC, USA; 5 Department of Epidemiology, Johns Hopkins Bloomberg School of Public Health, Baltimore, MD, USA; 6 Department of Statistics, University of Haifa, Mount Carmel, Haifa, Israel; 7 Department of Health Behavior, Gillings School of Global Public Health, University of North Carolina at Chapel Hill, Chapel Hill, NC, USA; 8 Instituto Dermatológico y Cirugía de La Piel, Santo Domingo, Dominican Republic; 9 Department of Prevention and Community Health, Milken Institute School of Public Health, George Washington University, Washington, DC, USA

**Keywords:** Mobility, Mobile, HIV/AIDS, Social capital, Caribbean, Community mobilization

## Abstract

Female sex workers (FSWs) are often a mobile population, but the role of mobility in influencing FSW social capital outcomes—defined here as engagement with HIV intervention activities and community mobilization—remains unclear. To date, the evidence is mixed, with mobility both facilitating and hindering community engagement, depending on the context. This study is the first to employ a mixed-methods approach to examine how mobility impacts these outcomes. We combined baseline survey data from 211 FSWs living with HIV with semi-structured interview data from a sub-cohort of 20 FSWs in Santo Domingo, Dominican Republic. Contrary to the expectation that mobility would reduce engagement, our quantitative findings indicate that mobile FSWs were more likely to participate in various HIV interventions, such as support groups for people living with HIV, compared to their non-mobile counterparts. Additionally, mobile women were more than twice as likely to engage in community mobilization efforts, such as rallies to promote sex worker rights, even after controlling for sociodemographic variables. Many participants, despite their mobility, shared that they actively engaged in HIV-related and community mobilization activities in Santo Domingo and gained benefits from these activities. Study implications highlight the need for more sex worker-driven activities in and outside of Santo Domingo to offer mobile women greater opportunities to connect and build social cohesion, as well as the development of mobile-friendly service delivery models and apps to facilitate connections when traveling outside the capital.

## Resumen

Las trabajadoras sexuales femeninas (TSF) suelen ser una población móvil, pero el papel de la movilidad en los resultados de capital social—definidos aquí como la participación en actividades de intervención en VIH y la movilización comunitaria—sigue siendo poco claro. Este estudio es el primero en emplear un enfoque de métodos mixtos para examinar este impacto. Combinamos datos de una encuesta basal de 211 TSF que viven con VIH con entrevistas semiestructuradas a un subgrupo de 20 TSF en Santo Domingo, República Dominicana. Contrario a lo esperado, los resultados cuantitativos indican que las TSF móviles tenían mayor probabilidad de participar en intervenciones de VIH y más del doble de probabilidad de involucrarse en esfuerzos de movilización comunitaria, incluso tras controlar variables sociodemográficas. Las implicaciones señalan la necesidad de más actividades lideradas por trabajadoras sexuales y de modelos de servicios y aplicaciones adaptados a la movilidad.

## Introduction

Mobility among female sex workers (FSWs) is often part of a livelihood strategy that allows women to support themselves and their families [1]. It has also been conceptualized as a form of capital, enabling FSWs to increase their income beyond what they would earn by staying in one location [2, 3]. Mobile FSWs strategically move to areas with greater market demand, such as tourist destinations or regions with seasonal workers, where they can earn higher wages [2, 4–6].

There is no consistent definition of mobility in the literature, and it is often used interchangeably with the term ‘migration.’ Bell and Ward [7] define mobility as any form of movement that is not permanent. Other researchers studying mobility among FSWs in low-to middle-income countries have conceptualized it as a type of ‘circulatory’ migration, which involves “moves back and forth between two locations, or at least between places and an original ‘home’” [5]. We define mobility as the temporary movement of individuals to specific locations, after which they return to their home after a set period of time [8]. Sex work-related mobility refers to temporary travel for the purpose of exchanging sex for money in locations away from one’s home (e.g., in another district or region) [5]. Estimates of mobile FSWs vary significantly, ranging from 11% in Vancouver, Canada, to 68% in Andra Pradesh, India [2, 9].

Drivers of mobility among women engaged in sex work are diverse, including new economic opportunities, subsistence needs, family obligations, and, in the case of FSWs with HIV, the desire to reduce stigma by seeking HIV services elsewhere to avoid being seen in their area of residence [2, 8, 10–12]. In the Dominican Republic, where this study was conducted, mobile FSWs are typically mothers with low levels of education, limited economic opportunities, and at least one child whom they often financially support on their own [13].

Mobility is also a key social determinant of health that impacts HIV prevalence among FSWs. HIV prevalence is above the global average for FSWs, with FSWs being 30 times more likely to be HIV-positive than women who are not FSWs [14]. In the Dominican Republic (DR), the HIV prevalence among FSWs is 4.2%, higher than the national HIV prevalence of 0.9% [15]. In terms of HIV prevalence among mobile FSWs, studies across different geographic and epidemic settings have shown that mobile FSWs have a higher prevalence compared to non-mobile FSWs. For example, in Andhra Pradesh, India, 68% of female sex workers were mobile, and 20% of mobile FSWs were HIV-positive, compared to 14.8% of non-mobile FSWs [9]. In South Africa, the HIV prevalence among mobile FSWs was 46.0%, compared to 34.7% among non-mobile FSWs [16].

Beyond its role in HIV acquisition, mobility also has important implications for engagement in HIV care and treatment among people living with HIV. A substantial body of literature documents how geographic mobility shapes HIV risk, care engagement, and treatment outcomes among people living with HIV. Mobility has been associated with disruptions across the HIV care cascade, particularly reduced retention in care and antiretroviral therapy (ART) adherence [17]. Qualitative and mixed-methods research from the Dominican Republic and transnational Dominican communities demonstrates that longer or unplanned travel, fear of HIV-related stigma at destinations, and structural constraints such as limited medication dispensing can contribute to treatment interruptions and delayed care seeking [17].

At the same time, the relationship between mobility and HIV care is not uniformly negative. Recent quantitative evidence from East Africa shows that different forms of mobility can have both harmful and protective effects on HIV care engagement, with outcomes varying by gender, travel motivation, and degree of control over mobility [18]. While migration may increase the risk of care discontinuation, some mobile individuals—particularly those with greater agency—develop adaptive strategies that allow them to maintain engagement in care or re-establish services in new locations [18].

Evidence from integrated HIV prevention interventions among sex workers in India [19, 20], Brazil [21], and the Dominican Republic [22] suggests that community empowerment interventions focusing on mobilizing communities and improving social capital (e.g., social support and social cohesion) can be effective in improving HIV prevention, treatment and care outcomes [23–25]. A key component of these interventions addressing HIV among FSWs is the social cohesion, solidarity, and mutual aid that exists within the sex worker community in a given setting. Social cohesion is generally understood as a critical first step in the community empowerment process, through which collective resources can be leveraged and community mobilization promoted to address socio-structural constraints on HIV outcomes [26].

Research on social capital outcomes resulting from HIV prevention interventions has not explored the role of mobility. Studies involving mobile women have yielded mixed results. For example, Davey et al. [26] found that increased mobility among FSWs in Zimbabwe was associated with higher attendance at community mobilization events. Other studies, however, have identified mobility as a barrier to FSW engagement in HIV prevention and care interventions [27, 28]. Febres-Cordero et al. [29] found that migrant sex workers at the Mexico-Guatemala border faced difficulties in establishing and accessing social networks and support, due to intersecting factors such as displacement, limited kinship networks, and experiences of stigma and discrimination in destination settings. A recent systematic review in Zimbabwe further demonstrated that these associations vary by context, highlighting the need for improved operationalization and measurement of mobility among FSWs [5]. As a result, it remains unclear whether mobility primarily undermines or facilitates engagement in HIV-related services and collective action. Calls for more context-specific, population-centered research emphasize the importance of understanding how mobile populations, including sex workers, navigate HIV care, stigma, and social networks across locations, and how health systems might adapt to better support them [30].

**Fig. 1:**
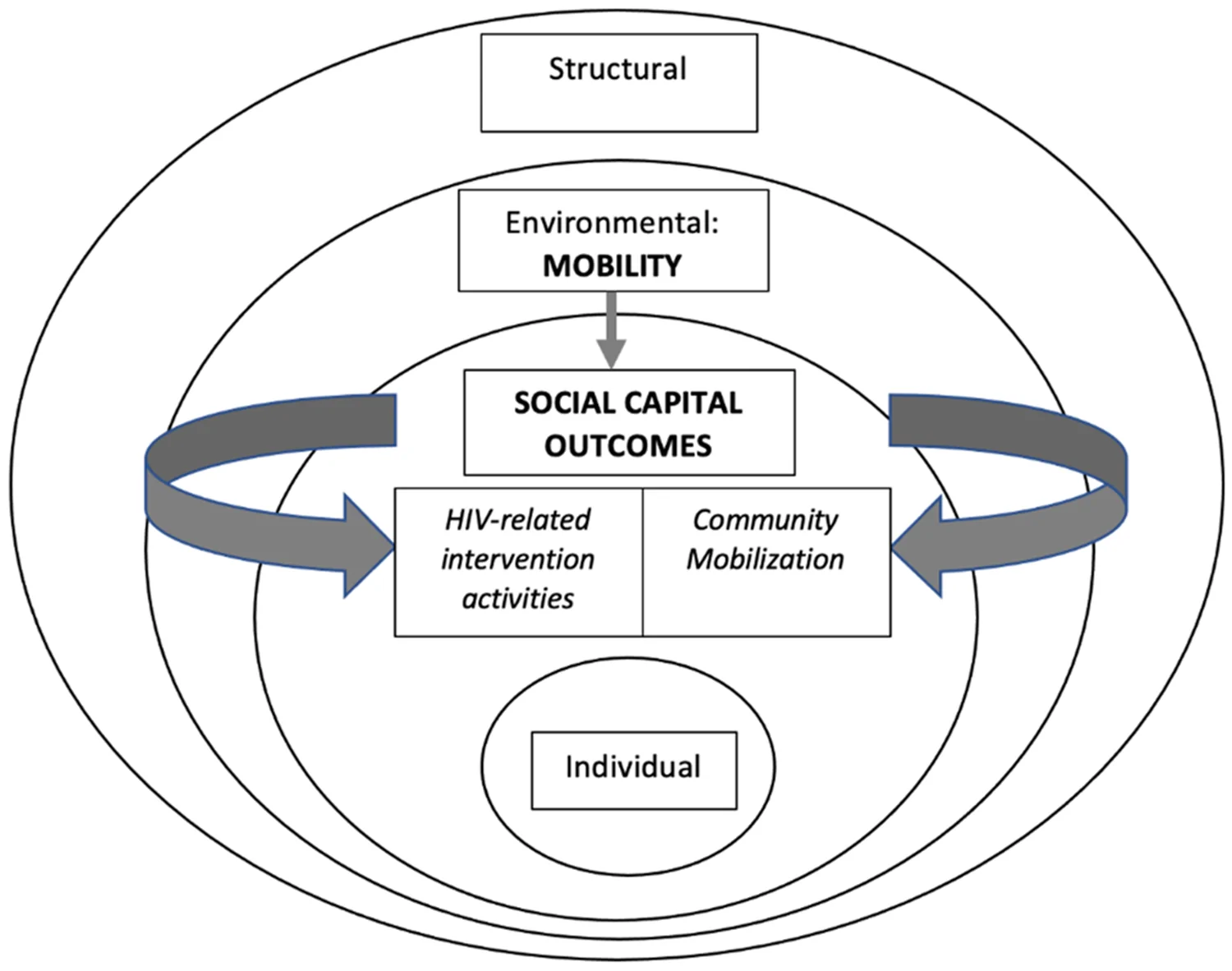
Theoretical framework of impact of mobility on social capital outcomes among female sex workers in Dominican Republic

To date, very few studies have examined the experiences of mobile female sex workers living with HIV in accessing and engaging with different forms of social capital, such as social cohesion and community mobilization activities. Research on this topic is critically important, as mobile FSWs living with HIV face unique challenges, including stigma and discrimination, lack of privacy and anonymity, and geographical barriers in accessing social support and participating in community empowerment initiatives [2, 31, 32]. Our study contributes to filling this gap by examining the relationship between mobility and social capital constructs—engagement with HIV-related intervention activities and community mobilization—among FSWs living with HIV in the Dominican Republic. Both of these constructs are conceptualized as output measures of social capital (Fig. 1).

## Social Capital Framework

We drew upon the social capital theory to frame the study. Social capital refers to the connectedness of individuals between and within groups [33]. Social capital theory was first defined by Bourdieu [34] as “the sum of the resources, actual or virtual, that accrue to an individual or a group by virtue of possessing a durable network of more or less institutionalized relationships of mutual acquaintance and recognition.” Since Bourdieu, Putnam has further refined the concept, defining it as “the networks, norms, and social trust that facilitate cooperation for mutual benefit” [35].

Social capital may be particularly important for FSWs because it provides both formal and informal opportunities for social cohesion and community mobilization, as well as access to material and social support resources that would otherwise be inaccessible to this population. FSWs comprise a group that faces heightened risk for HIV infection, exacerbated by social and structural factors, including poverty, gender inequality, sexual and physical violence, HIV and sex work-related stigma, discrimination, and marginaliztion [26, 37–40].

Guided by social capital theory [34,35], this study uses a mixed-methods design to examine the relationship between mobility and social capital outcomes among female sex workers living with HIV in the Dominican Republic. Drawing on quantitative survey data and qualitative interviews collected in Santo Domingo, we assess how mobility is associated with engagement in HIV-related intervention activities and participation in community mobilization. By situating these findings within the broader literature on mobility, HIV care, and sex work, this study aims to contribute context-specific evidence on how mobility shapes social capital among a highly mobile and structurally marginalized population.

## Methods

### Study Setting

The mixed-methods longitudinal cohort study, titled “Stigma, Cohesion, and HIV Outcomes Among Vulnerable Women Across Epidemic Settings,” integrated biological, survey, and qualitative data. It enrolled and followed a cohort of 416 FSWs in Santo Domingo, Dominican Republic (DR), and Iringa, Tanzania, who were 18 years or older, had a confirmed HIV-positive diagnosis, and reported exchanging sex for money in the month prior to enrollment [40]. Three study visits took place between 2016 and 2021. In this paper, we report on the data from Santo Domingo, integrating socio-behavioral quantitative survey findings with qualitative in-depth interview data (*N* = 211) to provide a holistic understanding of the role of mobility on the social capital variables among Dominican FSWs.

The research in the DR was conducted in partnership with a local research and HIV clinical service organization, the *Instituto Dermatológico y Cirugía de Piel* (IDCP), and the sex worker-rights group, *Movimiento de Mujeres Unidas* (MODEMU), located in Santo Domingo. Initial recruitment was primarily carried out by FSW *navegadoras* (peer navigators) from our community partner, MODEMU, who identified and contacted potential participants. This approach was supplemented by recruitment through clinics, key informants, and participants themselves. The study was approved by the Institutional Review Boards of the Johns Hopkins Bloomberg School of Public Health in the US, the IDCP, and the *Consejo Nacional de Bioética* (CONA-BIOS) in the DR. The study protocol was also reviewed by the IDCP Community Advisory Board. All participants provided informed consent and were compensated approximately USD $10 per study visit.

## Quantitative Measures

The socio-behavioral survey instrument included questions on individual, relational, environmental, and structural factors hypothesized to be related to HIV prevention, treatment behaviors, and outcomes in this population. We define our primary independent variable (mobility), dependent variables, and control variables below.

## Mobility

We combined two survey questions to measure mobility: FSWs were asked, “In the past 6 months, have you traveled outside of Santo Domingo?” and “In the past 6 months, have you traveled outside of Santo Domingo specifically to exchange sex for money elsewhere?”.

## Outcomes Related To Intervention Components

*Abriendo Puertas* (Opening Doors) is an integrated HIV care and prevention intervention conducted with FSWs with HIV in the Dominican Republic, targeting individual, relational, environmental, and structural barriers to both HIV prevention and treatment outcomes. The intervention components offer a multi-level response to the socio-structural context, as well as the stigma and discrimination experienced by FSWs living with HIV. *Abriendo Puertas* initial implementation and evaluation started in 2013 and project partners continued offering services through community based groups including the sex worker rights group, MODEMU. For the purposes of this article, we analyzed two social capital outcomes: engagement with HIV intervention activities and community mobilization. Table 1 describes the corresponding survey questions for these outcomes.

## HIV Intervention Engagement Outcome

We assessed engagement with HIV intervention activities, including passive forms of engagement (e.g., receiving HIV-related materials or seeing a poster about HIV/AIDS in various venues) and active forms (e.g., contact with peer educators or participation in group meetings at MODEMU).

## Community Mobilization Outcomes

We defined community mobilization as any action aimed at improving a group’s condition, in this case, the FSW community specifically [40]. It was measured by assessing participants’ engagement in formal groups (e.g., FSW-related meetings, marches, rallies, or gatherings to promote the health and rights of sex workers) or informal groups (e.g., get-togethers with other sex workers to address a common problem).

## Control Variables

We included variables to control for potential confounding factors: socio-demographics (age, education, number of children, relationship status, and income), sex work characteristics (years in sex work), and HIV-related factors (years living with HIV).

## Quantitative Data Analysis

We used descriptive statistics, including frequency distributions, medians, and ranges, to characterize the study sample (Table 2). We first examined bivariate relationships between mobility and the community mobilization and intervention activity engagement outcomes through simple logistic regressions. Subsequently, we investigated the same relationships in a multivariable logistic regression, adjusting for relevant socio-demographic, sex work, and HIV characteristics. We also checked for collinearity among the variables before inclusion. Results are shown as odds ratios (OR) and 95% confidence intervales. All analyses were conducted with STATA version 15 [41].

## Qualitative Data Collection

We recruited 20 women from the survey cohort to participate in a series of longitudinal in-depth interviews, usually conducted shortly after completing the survey for a given round. All interviews were conducted at the offices of the HIV Vaccine and Research Unit at IDCP. We purposively sampled based on viral load; half of the women in the qualitative sample were virally suppressed (≤400 copies/mL) and half were not (≥400 copies/mL). For the analysis, we used interview data from two time periods: baseline between October 2018 and November 2018 and during 12-month follow-up between December 2019 and March 2020. Baseline interviews lasted between 60 and 90 min and follow-up interviews lasting approximately 2 h. Interviews were conducted by a Dominican cisgender woman with a background in psychology and extensive experience conducting research with sex workers. Interviews were audio-recorded and transcribed verbatim and analyzed in Spanish.

We used a semi-structured interview guide that included country-specific questions, with a module focused on mobility over the 12-month period. In the first interview, experiences related to mobility emerged spontaneously as women discussed their engagement in sex work. In the follow-up interview, we included purposeful probing regarding participants’ mobility to deepen our understanding of how they attributed meaning to their mobility experiences. We also probed details about their preparations before traveling, their experiences during travel and at their destination, as well as their social relationships and HIV care-seeking behaviors, including ART adherence while traveling.

Sample mobility-related questions included a free-listing activity, where participants were asked to think about all the different places they typically visit both inside and outside of Santo Domingo, as well as an in-depth exploration of a typical trip they had made outside of the city. Interviewers probed for information on the following: where they went, the duration of the trip, preparations made before travel, details about the trip (including how they got there), difficulties encountered, the purpose of travel, social relationships at the destination, types of support received there, cell phone use, adherence to HIV care and treatment, and recommendations for future programs tailored to mobile FSWs.

## Qualitative Data Analysis

We employed a thematic analysis approach, which was iterative, data-driven, and inductive [42, 43]. Throughout the coding process, we used memo writing to document emerging themes, observations about the data, and our reflections on the significance and relationships of the codes, as well as to assist with data reduction and interpretation [42, 43].

Our memos also facilitated integration of themes over two different time periods (initial and follow-up interviews) to gain a deeper, more nuanced, and contextualized understanding of mobility and how the dynamic phenomenon influenced women’s participation in HIV intervention activities and community mobilization. We developed a codebook based on the themes emerging from the data. Using our memos, we synthesized the coding output across key domains and identified major themes using in vivo codes. These in vivo codes, which captured the essence of the participants’ experiences, preserved their exact language and the affective tone of their words, thus reflecting the authenticity and richness of their perspectives.

## Quantitative Results

Socio-demographic, behavioral characteristics, and social capital outcomes (HIV intervention activities and community mobilization) among FSWs living with HIV in Santo Domingo, Dominican Republic, are described in Table 2. The median age of participants was 40 years (range: 18–66), and about one-third (32.4%) were either married or living with a partner. A majority of participants (88.1%) had low levels of education (8th grade or less), had a mean monthly income of USD $173, and almost all women (93.0%) had children, with a median of 3 children.

Most women (94.5%) lived in the capital city of Santo Domingo, and many of them (80.6%) worked in a sex establishment or independently. The median number of years they had been engaged in sex work was 3. Although there was a wide range in the price that the women charged per client date (USD $1.46–$81.74), the median charge was approximately USD $9. Over a third of the sample (37.8%) reported alcohol use two or more times a week in the last 30 days. More than a third (36.3%) also reported ever having used drugs (e.g., marijuana, cocaine). The median number of years living with HIV was 9, and almost three-quarters (72.6%) of the sample were virally suppressed. Approximately a third of the women (32.2%) reported ever traveling outside of Santo Domingo in the past 6 months, with 65 (30.8%) reporting travel specifically for sex work.

Approximately a third of the women were actively engaged in HIV/AIDS-related intervention activities, such as participating in an HIV/AIDS educational activity or a support group for people living with HIV (32.8%) or contacting a peer educator about HIV/AIDS (38.3%). Activities not directly related to HIV/AIDS had lower attendance, such as workshops or group meetings at MODEMU (8.5%). In terms of community mobilization, almost one-quarter of the women (23.4%) reported participating in a meeting, march, rally, gathering, or organized group with other sex workers to promote their rights in the past 6 months.

In bivariate analysis (Table 3), FSWs who were mobile in the past 6 months were more likely to have participated in a meeting, march, rally, or gathering to promote the rights of sex workers compared to those who were not mobile in the past 6 months (OR = 2.28, *p* < .032). Mobile women were also significantly more likely to engage in organized groups with other sex workers in the past 6 months compared to non-mobile women (OR = 2.03, *p* < .021). Additionally, mobile FSWs were more likely to participate in HIV-specific activities in the past 6 months compared to non-mobile women, including HIV/AIDS educational activities (OR = 2.72, *p* < .001), support groups for people living with HIV (OR = 2.03, *p* < .018), and contact with a peer educator about HIV/AIDS (OR = 1.81, *p* < .048).

Mobile women were also significantly more likely than non-mobile women to be exposed to passive activities, such as receiving materials like a brochure about HIV/AIDS in the past 6 months (OR = 2.12, *p* < .001), having ever seen a poster about HIV/AIDS in their work venues (OR = 2.25, *p* < .012), receiving condoms from an HIV/AIDS organization in the past 6 months (OR = 2.62, *p* < .023), and receiving an SMS message related to HIV prevention and care in the past 6 months (OR = 3.76, *p* < .003).

In the multivariate logistic regression models (Table 3), mobility remained significantly associated with the same intervention activity engagement outcomes even after controlling for age, education, number of children, relationship status, income, years in sex work, and years living with HIV (AORs = 1.84 to 4.77, *p* < .050 and *p*< .001, respectively). Mobile women, compared to non-mobile women, were also slightly more than twice as likely to participate in community mobilization efforts, such as participating in an organized group, march, or rally to promote the rights of sex workers (AOR = 1.94 to 2.30, *p* < .050) after controlling for socio-demographic, sex work, and HIV variables.

## Qualitative Results

Consistent with the quantitative results, many participants, despite being mobile, shared how they actively engaged in HIV-related and community mobilization activities in Santo Domingo and benefited from these activities. Pseudonyms are used in the quotes below.

‘Being with these women is like being with family’

Most of the women who were mobile expressed receiving numerous benefits, including social cohesion and support, through the nurturing friendships they developed with other women by participating in HIV-related intervention activities. Isaura (29 years old), who traveled outside Santo Domingo for sex work, described how the relationships she formed with other women became a source of social support for her: “I learn a lot from the HIV group. I learn to live with my health condition, and I also learn to value the friendships that surround me, the other women in the group […] all of that helps me.” Guadalupe (31 years old), another mobile participant, had a similar sentiment: “Being with these women is like being with family, because I really feel very comfortable. I feel good. Sometimes I come with all the stress and all the problems from my house, and while I don’t forget the problems, in the group, I can get their help.”

Similarly, Carla (28 years old), who was mobile at least twice a month, also shared that she received social support from participating in the HIV support group, which contrasted with the alienation she felt from some individuals outside the group. She stated, “The women treat me well. They give me a hand to lift me up. Not like some people in the neighborhood who reject us.” Trina (36 years old), who traveled more frequently for work, shared a similarly positive group experience: “Right here is one of those groups. They give me a lot of support. They are the people I have always counted on, who have always given me affection and love. And if I need help, I know they will help me too. They always treat me with a lot of love, a lot of respect… It is that kind of group.”

‘*Everyone is real here*’.

Additionally, the results revealed that the HIV-related intervention activities offered a space for the participants to be their true selves. Women did not feel discriminated against in the intervention groups because everyone in the group shared an HIV-positive status and knew each other’s condition. The women felt accepted and supported in the group and by the peer educators. It was clear that mobility did not prevent them from engaging in and benefiting from the intervention groups. As Lucinda (24 years old), a participant with regular clients outside Santo Domingo, expressed, “Everyone is real here. I don’t know if it is because we are living the same condition [HIV status], but when we get together, we see that there is love…” Similarly, Sela (39 years old), who mentioned that she scheduled her travel to avoid missing the group meetings, shared positive sentiments about the group, “Oh, I feel like this is the only place where I can be authentic, because everyone who comes here knows my condition and I feel authentic… I don’t have to feel ashamed about having HIV, nor do I have to measure myself to say something because here we all know each other.” In the same vein, Carla (28 years old) recounted, “I have been in many of the groups with HIV-positive individuals. I feel confident. I am relaxed, open to talk, and say things that in another environment I could not say. I speak openly, without fear, because I know they are people just like me.”

‘I have learned many things’

Beyond the support and friendship, many mobile participants mentioned how they learned new information and skills, as well as experienced self-discovery, through participating in the HIV intervention activities. As Coralie (25 years old), a participant who mostly traveled to beach areas for sex work, related,

I belong to a group of *Chicas Lindas* that I like a lot. We are there participating and learning as much as possible. I have learned many things, from the other girls like making handicrafts and so many things, that I did not know that I had that capacity to do, to give, nor to receive. […] there are moments and people that come into your life that teach you and allow you to know yourself, to know things about you that you did not know you had to give.”

Similarly, Isaura (29 years old) shared, “Well, I feel good attending the workshops. Even my self-esteem changes. You always win there, you always feel good, because you always learn something. Even speaking there with other women, you feel it changes your mood, and you always get something new out of it.” Others shared how they also learned a lot from more passive HIV intervention activities, such as reading a brochure, tip card, or information sheet about HIV/AIDS from the women at MODEMU. Trina (36 years old) recounted, “The education on HIV that I’ve received is very good. I’ve learned from the materials they gave me to read at home. I dedicated myself to learning it. I learned how to stay undetectable and the importance of remaining undetectable, for my benefit and quality of life.”

‘Sharing like sisters’

Most women described the benefits of participating in meetings, marches, rallies, or gatherings to promote sex workers’ rights or address a common issue. Mobility did not seem to hinder their participation in or benefits from these activities. As Carla (28 years old) stated, “We would meet in different places, and it felt very good to talk to the women, to be part of a collective. We were all sharing like sisters. I felt happy when I was there with them.” Similarly, Lucinda (24 years old) shared, “For me, it was very important to go to those rallies. I learned a lot of things […] I learned to understand the others in the group and that they had problems like mine. The meetings taught me to understand the problems of other people, that it is not just me who has problems, that others also have similar problems.”

‘I feel empowered’

Getting involved in community mobilization activities also allowed the mobile participants to feel a sense of empowerment. Coralie (25 years old) described:

I started to get involved with the groups where people living with HIV meet. I have kept myself busy because those groups always have projects to improve the quality of life of people living with HIV. […] I feel empowered. I started talking to people who are not living with HIV and teaching them the importance of taking care of themselves and getting tested. I have worked on many projects, and I am active.

Sela (39 years old) also shared, “I have tried to learn as much as I can about many of the people who have been living with HIV for many years. I have gotten together with them […] For me, it is about feeling empowered and having a better quality of life living with HIV.

Overall, participants described these HIV-related and community mobilization activities as safe, supportive, and more accepting than other environments. Many noted that their mobile experiences in nearby towns, resort areas, or larger cities involved stigma or discrimination. For instance, several women avoided locations where they had previously faced judgment or harassment, while others planned travel to maintain access to peer-led support groups and HIV services.

## Discussion

This study sheds light on the relationship between mobility and social capital outcomes among FSWs living with HIV in the Dominican Republic. Contrary to expectations that mobility might decrease engagement with HIV interventions and community mobilization, our quantitative findings suggest that mobile FSWs were more likely to participate in these activities than their non-mobile counterparts. These findings demonstrate that mobile FSWs had significantly higher odds of engaging in HIV intervention activities, such as receiving educational materials and participating in support groups, respectively. Moreover, mobile women were more likely to partake in community mobilization efforts, including rallies and advocacy meetings for sex worker rights. These results indicate that mobility does not inherently hinder social capital development but may, in fact, serve as a catalyst for engagement in HIV-related activities, community-based support networks, and community mobilization.

The observed paradox—where increased mobility correlates with higher engagement in social capital activities—may be explained by the unique challenges faced by mobile FSWs. Mobility can disrupt social networks at both home and destination locations, compelling women to actively seek out opportunities to maintain their support systems. Many participants described how structured HIV interventions, such as those provided by MODEMU, offered a unique space where they felt free of judgment and stigma. This finding underscores the importance of creating inclusive, community-driven spaces that foster social cohesion among FSWs, particularly those who experience heightened vulnerability due to mobility.

Additionally, these findings align with previous research suggesting that exposure to stigma and discrimination at destination locations may drive mobile FSWs to seek out and strengthen social support networks upon their return home [29]. The findings are also consistent with Davey et al.’s [26] study, which reported that greater mobility among FSWs in Zimbabwe was linked to increased participation in community mobilization events. The qualitative data further support this interpretation, with participants highlighting the emotional and practical benefits of engagement in peer-led HIV activities, which provided a sense of belonging, empowerment as well as access to vital health information and new skills.

Moreover, consistent with social capital theory, our findings suggest that mobility can be conceptualized as a form of agency and an adaptive strategy that reshapes how individuals interact with support networks and information. Mobile women actively sought out, maintained, or intensified connections to supportive environments, including engagement in passive intervention activities (e.g., exposure to printed materials, posters, SMS), in response to heightened vulnerability, stigma, or service disruption associated with travel. Taken together, these narratives suggest that mobility is also shaped by the need to navigate stigmatizing social environments, underscoring stigma as a key factor influencing movement patterns.

However, other studies present contrasting findings, suggesting that mobility can act as a barrier to FSW engagement in HIV prevention and care interventions [27, 28]. A recent systematic review in Zimbabwe highlighted how these associations can vary by context, emphasizing the need for improved conceptualization and measurement of mobility among FSWs [5].

This study has several strengths. First, the mixed-methods approach allowed for a comprehensive analysis by integrating quantitative and qualitative data, which provided depth and context to the statistical findings. Second, the study benefits from a strong community partnership with MODEMU, ensuring that the research was grounded in the lived experiences of FSWs. Third, this study allowed us to longitudinally examine the role of mobility in HIV intervention engagement and community mobilization among women living with HIV. Fourth, the study’s focus on mobile FSWs fills an important gap in the literature, offering new insights into how mobility influences engagement with HIV interventions and community mobilization. Fourth, missing piece?

However, there are also limitations. The sample was primarily drawn from FSWs with access to an established FSW organization, potentially introducing selection bias, as those who are already connected to support networks may have different experiences from those who are not. Additionally, the reliance on self-reported data introduces the possibility of recall and social desirability biases, which may affect the accuracy of responses regarding mobility patterns and engagement in interventions. Another limitation of this study is the broad operationalization of mobility. Future research should incorporate more nuanced mobility measures to better distinguish how different forms of movement relate to engagement in HIV interventions and community mobilization. Studies with larger qualitative samples are also needed to explore how the purpose, duration, and frequency of mobility, as well as differences between mobile and non-mobile FSWs, influence experiences of HIV care and community engagement. Lastly, several outcomes had relatively small cell sizes, which may limit statistical power and increase uncertainty around some adjusted estimates. Covariates were selected a priori based on theory and prior literature, and future studies with larger samples are needed to confirm these associations.

## Implications for Policy and Practice

The findings of this study have important implications for the design and implementation of interventions targeting mobile FSWs with HIV. Firstly, intervention programs should be tailored to acknowledge and leverage the strengths of mobile FSWs. Rather than viewing mobility as a barrier, policies should integrate mobile-friendly service delivery models, including pop-up health clinics [44, 45], mobile phone-based apps and programs [46, 47], peer outreach in key transit areas [48, 49], and flexible HIV care options that accommodate movement patterns. Lessons can be drawn from other examples, such as the Sisters Clinics in Zimbabwe, which provide decentralized, low-barrier access to HIV care for mobile FSW populations [50]. Another example in the Netherlands is the Amsterdam Center for Sex Workers, which is dedicated to empowering and supporting sex workers in the Amsterdam region by providing free and anonymous healthcare and advice while upholding their right to freely choose and practice their occupation without exploitation [51].

Secondly, advocacy efforts should focus on legal protections and anti-stigma campaigns that address the discrimination faced by mobile FSWs [52]. Strengthening legal frameworks that protect sex workers’ rights and reducing institutionalized stigma can improve access to healthcare and social support services [53]. Drawing from the regulatory models of countries like the Netherlands and Germany, where sex work is legally recognized, could offer insights into policies that enhance the well-being and agency of FSWs. For instance, Germany has a legal framework that recognizes sex workers as legal employees, requires regular health checks to support their well-being, and provides access to sex work counseling resources [54]. Thirdly, future research should distinguish between sex work–related mobility from other forms of travel, as these distinct mobility patterns may differentially influence HIV service engagement, social capital formation, and community mobilization among FSWs living with HIV. Moreover, future studies should assess whether interventions tailored to mobile FSWs living with HIV expand their social networks beyond their home communities, leading to sustained improvements in HIV outcomes and social cohesion.

## Conclusion

Our findings highlight that mobile FSWs were more likely to actively seek opportunities to engage with HIV interventions and community mobilization compared to their nonmobile counterparts, demonstrating that mobility was not a barrier. A plausible explanation is that mobile women increased their engagement as a strategy to strengthen and maintain their support systems and counteract the negative effects of mobility-related challenges. Policymakers and practitioners should build on these findings to design interventions that are responsive to the dynamic realities of mobile FSWs, ensuring that they remain connected to support networks and health services regardless of their location. Expanding peer-led, mobile-friendly, and legally protective interventions will be key to improving HIV outcomes and collective empowerment among this population.

## Figures and Tables

**Table 1:** Social capital outcome variables and corresponding survey items

HIV intervention activities
Passive Activities
-Received any materials like a brochure, tip card, or information sheet about HIV/AIDS in the past 6 months
-Ever seen a poster about HIV/AIDS in any of the venues where you worked in the past 6 months
-Received condoms from a HIV/AIDS organization in the past 6 months
-Received an SMS text message related to HIV prevention and care in the past 6 months
Active Activities
-Attended a workshop at the MODEMU in the past 6 months
-Participated in a group meeting at the MODEMU in the past 6 months
-Had contact with a peer educator/navigator about HIV/AIDS in the past 6 months
-Participated in a support group with other people living with HIV in the past 6 months
-Participated in individual counseling about living with HIV in the past 6 months
-Participated in an educational activity about HIV/AIDS in the past 6 months
Community Mobilization
Formal Groups
-Participated in a meeting, march, rally or gathering to promote the rights of sex workers in the past 6 months
-Participated in an organized group with other sex workers in the past 6 months
Informal Groups
-Gotten together with other sex workers to speak with government officials or political leaders to address a problem or common issue facing sex workers in the past 6 months
-Joined together with other sex workers to address a common problem facing sex workers in the past 6 months

**Table 2:** Socio-demographic, behavioral characteristics, community mobilization outcomes, and intervention activity engagement outcomes among FSWs living with HIV in Santo Domingo, Dominican Republic (*N* = 211)

Variables	Percentage (Frequency) in Each Category	Median (Range/ Interquartile Range)
Socio-demographic and behavioral characteristics		
Age (years)		41 (21–67; 11)
Civil status		
Single/Never married	49.8% (105)	
Married with papers and living together	1.9% (4)	
Living with someone without papers	39.3% (83)	
Separated	5.7% (12)	
Widowed	3.3% (7)	
Education level		
0-8th grade	61.1% (129)	
9th grade to university graduate	38.9% (82)	
Mean monthly income (USD $)^a^		173
Has any children	94.3% (199)	
Number of children		3 (0–9; 2)
Current residence		
Santo Domingo	85.8% (181)	
Another city/town/rural area	14.2% (30)	
Type of sex work venue		
Street	26.5% (56)	
Sex establishment or independent	73.5% (155)	
Years in sex work		5 (0–45; 8)
Average price per date in pesos (and in USD)		500 (0-3666.67; 700.00) $8.76 ($0-$64.22; $12.26)
Alcohol use in the last 30 days		
Never	18.0% (38)	
Once a month or less	23.2% (49)	
2 to 4 times a month	24.2% (51)	
2 to 3 times a week	21.8% (46)	
4 or more times a week	12.8% (27)	
Drug use ever	26.5% (56)	
Years living with HIV		10 (1–41; 9)
Viral suppression (< 400) Mobility (independent variable)	75.8% (160)	
Ever traveled outside of Santo Domingo in the past 6 months for sex work or other reason	32.2% (68)	
HIV intervention activity engagement outcomes		
Passive Activities		
Received any materials like a brochure, tip card, or information sheet about HIV/AIDS in the past 6 months	50.7% (107)	
Ever seen a poster about HIV/AIDS in any of the venues where you worked in the past 6 months	61.1% (129)	
Received condoms from a HIV/AIDS organization in the past 6 months	78.7% (166)	
Received an SMS text message related to HIV prevention and care in the past 6 Months	11.9% (25)	
Active Activities		
Attended a workshop at the MODEMU in the past 6 months	9.5% (20)	
Participated in a group meeting at the MODEMU in the past months	10.4% (22)	
Had contact with a peer educator/navigator about HIV/AIDS in the past 6 months	38.9% (82)	
Participated in a support group with other people living with HIV in the past 6 months	41.2% (87)	
Participated in individual counseling about living with HIV in the past 6 months	55.5% (117)	
Participated in an educational activity about HIV in the past 6 months Community mobilization outcomes	54.5% (115)	
Formal Groups		
Participated in a meeting, march, rally, or gathering to promote the rights of sex workers in the past 6 months	15.6% (33)	
Participated in an organized group with other sex workers in the past 6 months	33.2% (70)	
Informal Groups		
Gotten together with other sex workers to speak with government officials or political leaders to address a problem or common issue facing sex workers in the past 6 months	7.1% (15)	
Joined together with other sex workers to address a common problem facing sex workers in the past 6 months	34.6% (73)	

a Dominican pesos were converted to U.S. dollars (US$) based on the conversion rate of 0.018 dollars per DR peso

**Table 3:** Unadjusted and adjusted odds of community mobilization and intervention activity engagement outcomes among FSWs living with HIV in the DR by mobility in the last 6 months as an independent Variable^a^

HIV intervention activity engagement outcomes	OR	95% CI	*p*-value	AOR	95% CI	*p*-value
Passive Activities						
Received any materials like a brochure, tip card, or information sheet about HIV/AIDS in the past 6 months	2.12	1.173 to 3.842	0.13*	1.99	1.082 to 3.688	0.027*
Ever seen a poster about HIV/AIDS in any of the venues where you worked in the past 6 months	2.25	1.197 to 4.232	0.012*	2.22	1.138 to 4.338	0.019*
Received condoms from a HIV/AIDS organization in the past 6 months	2.62	1.145 to 5.987	0.023*	2.50	1.045 to 5.967	0.039*
Received an SMS text message related to HIV prevention and care in the past 6 months	3.76	1.591 to 8.906	0.003**	4.77	1.839 to 12.369	0.001**
Active Activities						
Attended a workshop at the MODEMU in the past 6 months	1.46	0.566 to 3.746	0.436	1.12	0.402 to 3.111	0.831
Participated in a group meeting at the MODEMU in the past months	1.53	0.618 to 3.766	0.360	1.35	0.519 to 3.534	0.535
Had contact with a peer educator/navigator about HIV/AIDS in the past 6 months	1.81	1.005 to 3.256	0.048*	1.84	1.005 to 3.369	0.048*
Participated in a support group with other people living with HIV in the past 6 months	2.03	1.129 to 3.648	0.018*	1.86	1.011 to 3.420	0.046*
Participated in individual counseling about living with HIV in the past 6 months	1.34	0.745 to 2.406	0.330	1.32	0.718 to 2.411	0.375
Participated in an educational activity about HIV in the past 6 months	2.72	1.470 to 5.043	0.001***	2.75	1.461 to 5.162	0.002**
COMMUNITY MOBILIZATION OUTCOMES						
Formal Groups						
Participated in a meeting, march, rally or gathering to promote the rights of sex workers in the past 6 months	2.28	1.072 to 4.853	0.032*	2.30	1.043 to 5.059	0.039*
Participated in an organized group with other sex workers in the past 6 months	2.03	1.113 to 3.712	0.021*	1.94	1.030 to 3.649	0.040*
Informal Groups						
Gotten together with other sex workers to speak with government officials or political leaders to address a problem or common issue facing sex workers in the past 6 months	0.75	0.230 to 2.447	0.634	0.69	0.199 to 2.352	0.547
Joined together with other sex workers to address a common problem facing sex workers in the past 6 months	0.86	0.468 to 1.592	0.637	0.76	0.400 to 1.459	0.415

* *p* < .05

** *p* < .01

*** *p* < .001

a Controlling for age, education, number of children, relationship status, income, years in sex work, and years living with HIV for all outcomes. *AOR* Adjusted odds ratio

## Data Availability

The dataset for the current study is available from the corresponding author, Dr. Maria De Jesus, via an email request (dejesus@american.edu) due to adherence to privacy and confidentiality agreements.
